# Quantitative and visual analyses of the effect of activity reduction on image metrics and quality in 18F-FDG PET/MRI in pediatric oncology

**DOI:** 10.55730/1300-0144.5584

**Published:** 2022-07-18

**Authors:** Uğuray AYDOS, Erdem BALCI, Seda GÜLBAHAR ATEŞ, Ümit Özgür AKDEMİR, Ceyda KARADENİZ, Lütfiye Özlem ATAY

**Affiliations:** 1 Department of Nuclear Medicine, Faculty of Medicine, Gazi University, Ankara, Turkey; 2 Department of Pediatric Oncology, Faculty of Medicine, Gazi University, Ankara, Turkey

**Keywords:** PET/MRI, ^18^F-FDG, pediatric, tracer activity, texture analysis

## Abstract

**Background/aim:**

The aim of our study was to evaluate the effect of reduced injected tracer activities on the quantitative image metrics and the visual image quality in whole-body ^18^F-FDG PET/MRI with TOF capability in pediatric oncology.

**Materials and methods:**

Seventy-seven PET/MRI examinations of 54 patients were analyzed (standard injected activity: 1.9 MBq/kg, standard PET scan duration: 5 min per bed position). Lower activity PET images (1.2 MBq/kg and 0.9 MBq/kg) were retrospectively simulated from the originally acquired list-mode data sets. Quantitative parameters were assessed by measuring the SUV metrics, signal-to-noise ratio (SNR), contrast-to-noise ratios (CNR), and textural features in each PET data set. PET images were also evaluated visually for image quality by using a scoring system.

**Results:**

SNRs were found as significantly different among PET data sets (p < 0.001) and showed increasing image noise with decreasing activities. CNR values did not show significant differences among PET data sets. The mean relative percentage changes in SUV metrics were found to be lower in 1.2 MBq/kg data set compared to 0.9 MBq/kg data set. Lesion SUVmax, SUVmean, SULpeak, and textural features were significantly different in 0.9 MBq/kg data set compared to the original data set (p < 0.05 for all). However, SUV metrics and textural features did not show a significant difference between the original and 1.2 MBq/kg data sets. While, the mean visual scores in 0.9 MBq/kg data set were significantly different compared to the original data set (p < 0.001), there was no significant difference between the original and 1.2 MBq/kg data sets in terms of general image quality and image sharpness.

**Conclusion:**

Our analyses showed that the reduction of injected activity to 1.2 MBq/kg may be feasible in pediatric oncological PET/MRI, with a smaller relative percentage change in quantitative parameters and with similar image quality to the original data set.

## 1. Introduction

Fluorine-18 fluorodeoxyglucose (^18^F-FDG) positron emission tomography/computed tomography (PET/CT) is a routinely used imaging modality for primary staging, evaluation of therapy response, and restaging of various malignancies in pediatric oncology [1–4]. However, repeatedly performed PET/CT imaging during follow-up may increase the cumulative ionizing radiation exposure and the risk of radiation-induced long term adverse events in pediatric patients [5, 6].

Hybrid PET/magnetic resonance imaging (MRI), which is a prominent modality in pediatric oncological imaging in recent years, is important in terms of radiation exposure since MRI does not contain ionizing radiation, in addition to its advantages such as simultaneous imaging, higher soft tissue contrast, and the opportunity of functional imaging [7, 8]. In particular, PET/MRI can provide a significant advantage in the pediatric age group, because they are more radiosensitive than adults and more prone to radiation-induced long-term adverse effects, including radiation-induced malignancies [9,10].

Radiation exposure can be significantly reduced by up to 70% by replacing CT with MRI using hybrid PET/MRI scanners [11]. In addition to the elimination of CT-related radiation dose, the higher sensitivity of solid-state PET detectors in current PET/MRI scanners and the possibility to extend PET acquisition times due to the simultaneous acquisition of PET and MRI make it possible to further reduce the radiation exposure by decreasing the injected radiotracer activities [12–15].

To our knowledge, there have been limited number of published studies to date that evaluated the effect of lower injected activities on visual image quality and quantitative image metrics in ^18^F-FDG PET/MRI in pediatric oncology [16–18]. As a result of the visual and quantitative analyses in these previous studies, it has been shown that a proportional reduction of injected tracer activity can be feasible in pediatric oncological PET/MRI. However, these studies were conducted by using different PET/MRI scanners and different PET acquisition times per bed position. While two of these studies used similar commercial PET/MR systems (Biograph mMR, Siemens), one recent study was conducted by using a different PET/MR scanner (SIGNA, GE Healthcare) with time-of-flight (TOF) capability and higher photon sensitivity [16–19]. For this reason, it should be taken into account that the different results of these previous studies (minimum acceptable activity amounts were 1.5 MBq/kg and 1.2 MBq/kg, respectively) can be related to the properties of different scanners and different PET acquisition times. Furthermore, previous studies were conducted with relatively small patient populations and they did not examine the effect of tracer activity reduction on textural features. Further studies with larger patient populations are needed in this field.

Our hospital has installed a simultaneous PET/MRI scanner (GE SIGNA) in December, 2015. From December 2015 to September 2020, a total of 482 pediatric imaging with ^18^F-FDG PET/MRI were performed at our department. Three hundred and eighty-two of these imaging studies were performed for oncological investigation. The first 130 oncological PET/MR imaging studies were acquired by using a dosing regimen of 3.7 MBq/kg of ^18^F-FDG. The reduction of the standard injected activities to 1.9 MBq/kg with 5 min of PET acquisition time per bed was planned based on the previous study by Gatidis et al. [16]. Based on our clinical experience and the joint decision taken with the Pediatric Oncology Department, the standard injected activity in pediatric oncology patients imaged with PET/MRI has been reduced to 1.9 MBq/kg since September 2017 [8]. Totally, 252 oncological PET/MRI studies were performed by using 1.9 MBq/kg of ^18^F-FDG activity as a clinical standard activity until September 2020.

The aim of our study was to evaluate the effect of reduced injected tracer activities on the quantitative image metrics, radiomics features, and the visual image quality in whole-body ^18^F-FDG PET/MRI with TOF capability in pediatric oncology. The present study was conducted to examine whether the injected tracer activity could be further reduced from 1.9 MBq/kg to lower levels in pediatric oncological PET/MRI studies.

## 2. Materials and methods

### 2.1. Patients

This study was a retrospective, cross-sectional study. A total of 77 pediatric oncological ^18^F-FDG PET/MRI examinations of 54 patients (25 girls, 29 boys; mean age 10.3 ± 4.9 [2–18] years) performed between November 2019 and September 2020 in Gazi University, Medical Faculty, Nuclear Medicine Department were used. Pediatric patients who a) were followed by Pediatric Oncology Department, b) underwent FDG PET/MR imaging in Nuclear Medicine Department and c) had retrospectively simulated lower tracer activity images were included in the study. Patients who a) were older than 18 years and b) did not have simulated lower activity images were excluded from the study. Fifteen patients needed sedation for PET/MRI scan. Forty-eight patients had diagnosed malignancies (19 sarcoma, 15 Hodgkin’s lymphoma, 5 neuroblastoma, 2 leukemia, 2 germ cell tumor, 1 Burkitt lymphoma, 1 adrenal cortical tumor, 1 malign melanoma, 1 lung mucoepidermoid carcinoma, and 1 Wilm’s tumor). Five patients were diagnosed with Langerhans cell histiocytosis and one patient was diagnosed with infantile myofibromatosis. Of 77 PET/MRI examinations, 17 were performed for primary staging, 39 for treatment response evaluation, and 21 for restaging. This study was approved by the local Ethics Committee of our university (date: 10 February 2020, decision number: 147), and the need for written informed consent was waived.

### 2.2. PET/MRI examination

All PET/MRI acquisitions were performed on a 3-Tesla hybrid PET/MRI scanner with TOF capability (GE SIGNA PET/MR, GE Healthcare, Waukesha, Wisconsin, USA). Patients were injected with 1.9 MBq/kg of ^18^F-FDG. All patients fasted at least 6 h before the PET/MRI examination. The mean uptake time of all patients was 59.2 ± 12 min. All PET data were acquired and stored in list mode with 5 min per bed position. PET/MRI acquisition from vertex-to-toes was performed for all patients. The mean total acquisition time was 34.5 ± 7.7 min.

Whole body PET/MRI protocol included an initial localizer scan and a 3D dual-echo fast spoiled gradient recalled echo liver-accelerated volume acquisition sequence (LAVA-FLEX) for MRI based attenuation correction. Whole body PET/MRI also included high resolution axial T1-weighted 3D LAVA-FLEX sequence, coronal T2-weighted fast-recovery fast spin echo (FRFSE) sequence, coronal T2-weighted short-tau inversion recovery (STIR) sequence, whole body diffusion weighted images (DWI, b values: 50, 1000 s/mm^2^), and apparent diffusion coefficient (ADC) mapping. Additional sequences without contrast enhancement were acquired in some patients, depending on the diagnosis and previous imaging. For the attenuation correction, an atlas-based attenuation correction map was used for the head and vendor-provided algorithm using MRI-based attenuation correction data was used for the remaining body parts.

### 2.3. Data reconstruction and simulation of reduced-activity PET images

Acquisitions were reconstructed by using TOF-Ordered Subset Expectation Maximization (OSEM) algorithm with 2 iterations and 28 subsets, 192 × 192 image matrix, a voxel size of 2 mm, a 5 mm filter, standard scatter correction and without point-spread-function modeling.

Reduction of administered tracer activity from 1.9 MBq/kg (original data set) to 1.2 MBq/kg and to 0.9 MBq/kg were simulated retrospectively in all patients by truncating the original list mode data at 3 min 20 s and 2 min 30 s for bed position, respectively. For each examination, three result datasets, including the original dataset, were reconstructed and 231 image volumes were analyzed in total.

### 2.4. Quantitative analysis

All PET data sets were included for the quantitative analysis. PET data sets were analyzed by using vendor-based workstation (AW Volume Share 5, GE, Medical Systems). In order to examine the influence of activity reduction on quantification, volumes of interest (VOIs) were placed within physiological FDG uptake sites (liver, mediastinal blood pool [MBP], bone marrow at the body of L4 vertebra level [BM], psoas muscle and urinary bladder) and FDG-avid lesions. VOIs in liver had diameters of 3 cm, and in the other sites VOIs had diameters of 2 cm. VOI diameter was reduced to 1 cm within MBP in smaller pateints. VOIs covering FDG-avid lesions were defined as a 42%-isocontour of the maximum ^18^F-FDG uptake within the lesion. VOI positions and sizes were selected on the original data set and copied to other simulated data sets to obtain the same VOI size and localization. Objective quantitative parameters were assessed by measuring the maximum and mean standardized uptake values (SUVmax, SUVmean) as well as SUV variations (SUVvar) in physiological sites and FDG-avid lesions in each PET data set. SUVmax was defined as the maximum voxel value within the VOI. SUVmean was defined as the average of voxel values within the VOI. SUVvar was defined as the standard deviation of the SUV of all voxels within the VOI. In each PET data set, a maximum of 5 lesions with higher FDG-uptake than the liver and the one lesion with the lowest FDG uptake were selected for quantification. If there were more than 5 lesions with a higher FDG uptake than the liver, the first 5 lesions with the highest FDG uptake were selected. Thus, SUV measurements were performed on a maximum of six FDG-avid lesions in each PET/MRI data.

In addition, another quantitative analysis of FDG-avid lesions by using lean body mass corrected SUV (SUL) peak parameter was performed according to PERCIST (Positron Emission Tomography Response Criteria in Solid Tumors) in order to determine the effect of activity reduction on clinical oncological studies. For this purpose, liver threshold values were calculated by using the formula of [1.5 × liver SULmean] + [2 × standard deviation]. A maximum of 5 malignant lesions with the higher SULpeak than liver threshold was selected for the quantitative analysis. SULpeak levels were measured from these FDG-avid target lesions in each PET data set. The SULpeak was defined as the average of voxel uptake values of a 1 cm^3^ spheric VOI centered on the highest uptake part of the tumor [20, 21].

Signal-to-noise ratio (SNR), as a quantitative evaluation of image quality, was calculated as the liver SUVmean divided by the liver SUVvar by using the liver VOI in each PET data set. In addition to this, contrast-to-noise ratios (CNR) of the FDG-avid lesions with the maximum uptake (CNRmax) and with the minimum uptake (CNRmin), as objective metrics of lesion detectability, were calculated in each PET data set as defined in the formula: (Lesion SUVmean–background SUVmean)/(background standard deviation). Background regions were defined as the outer shell of lesion VOI and two voxels in size [17, 22].

### 2.5. Visual evaluation of image quality

PET data sets of the patients were ordered randomly by removing the patient names and data set information. PET images were displayed in axial, coronal, sagittal orientations. These images and MIP images were visually evaluated by one nuclear medicine specialist without seeing MR images. PET images were scored by the reader according to the general image quality and artifacts, image sharpness, image noise, lesion detectability using a 4-point scale (1: excellent/clear, 2: good, 3: average, 4: inadequate/poor) [23].

### 2.6. Texture analysis

**Figure 1:**
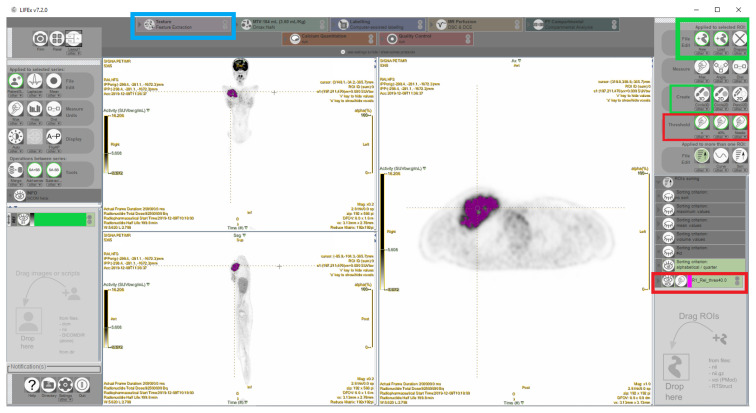
Texture analysis of ^18^F-FDG PET images using software (LIFEx 7.2.0). A primary tumor was seen in the right pectoral region on coronal (left top), sagittal (left bottom), and axial (right) FDG PET images. Volume of interest (VOI) (purple) was delineated around the tumor by using a circular 3D VOI tool (green boxes on the right panel). Primary tumor lesions were segmented by using 40% of the maximum value in the VOI as a threshold (red boxes on the right panel). Texture features were extracted from tumor VOIs using Texture Feature Extraction section (blue box on the top panel).

Texture analyses of FDG PET images of three data sets were performed in fourteen patients with primary solid tumors (9 sarcoma, 2 germ cell tumor, 1 neuroblastoma, 1 adrenal cortical tumor, 1 lung mucoepidermoid carcinoma) to evaluate the effect of tracer activity reduction on quantitative radiomics features. For each patient, axial, coronal and sagittal PET images were processed into DICOM (Digital Imaging and Communications in Medicine) format. For the textural analysis, the LIFEx version 7.2.0 software was used [24].{Nioche, 2018 #635} VOIs were delineated around the gray-scale images of primary tumors by using circular three-dimensional (3D) VOI tools of this program by one nuclear medicine specialist. These VOIs were used for the radiomic feature extraction of histogram, grey level cooccurence matrix (GLCM), grey-level run length matrix (GLRLM), neighborhood grey-tone difference matrix (NGTDM) and grey-level size zone matrix (GLSZM) analyses in each PET data set. Primary tumor lesions were segmented by using 40% of the maximum value in the VOI as a threshold (Figure 1). For intensity discretization, a bin size of 0.3125 and a grey-level number of 64.0 were used. For absolute intensity rescaling, SUV intensity from 0.0 to 20.0 was used. These are the default settings of the software and have not been changed in our study. The histogram analysis makes a global assessment of voxel intensity within the image. The GLCM is computed from the arrangements of pairs of voxels from 13 different directions and demonstrates the relationship between neighboured voxels. The GLRLM analysis is performed by calculating the size of homogeneous runs for each grey level and is computed for the 13 different directions. The 3D index value for both GLCM and GLRLM is the average of the index over the 13 directions. NGTDM demonstrates the difference of grey-level between one voxel and its 26 neighbours in 3D format. GLSZM provides information on the size of homogeneous zones for each grey-level in 3 dimensions.

### 2.7. Statistical analysis

Differences in SUV quantitative parameters of simulated data sets were also recorded as relative percentage changes compared to the original PET data set. Nonparametric Friedman test was used to statistically compare the quantitative measurements and visual scores between PET data sets with post hoc analyses for pairwise comparisons. Statistical analyses were performed by using the IBM SPSS Statistics for Windows (version 23.0, IBM Corp., Armonk, New York) software. For all analyses, a p value of <0.05 was considered statistically significant. Statistical power analyses of the variables in the study were performed considering sample sizes, effect sizes and statistical significance level (0.05). The effect sizes of each variable were calculated by using Kendall’s W value in the Friedman test.

## 3. Results

### 3.1. Quantitative analyses

**Figure 2:**
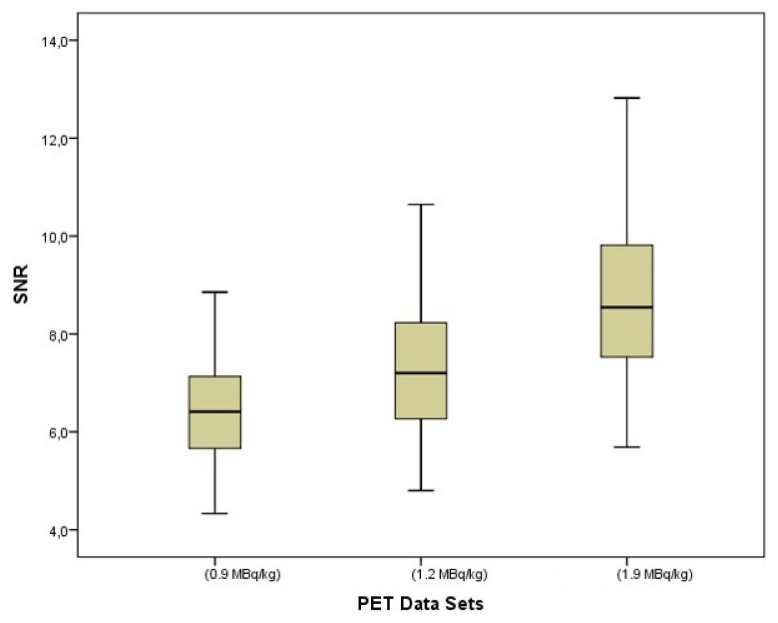
The boxplot of signal-to-noise ratios (SNR) among PET data sets. SNRs were found as significantly different among PET data sets (p < 0.001) and showed gradually increasing image noise with decreasing activities.

SNRs were found as significantly different among PET data sets (p < 0.001) and showed gradually increasing image noise with decreasing activities. The median values of SNRs were 8.5 (5.7–12.8) at the original data set, 7.2 (4.8–10.7) in the 1.2 MBq/kg data set, and 6.4 (4.3–8.9) in the 0.9 MBq/kg data set. In pairwise comparisons, significant differences in median values of SNRs were seen between PET data sets (p < 0.001 for all) (Figure 2). The power analysis result of the SNR for the sample size used (n = 77) was 1.0.

**Figures 3a and 3b:**
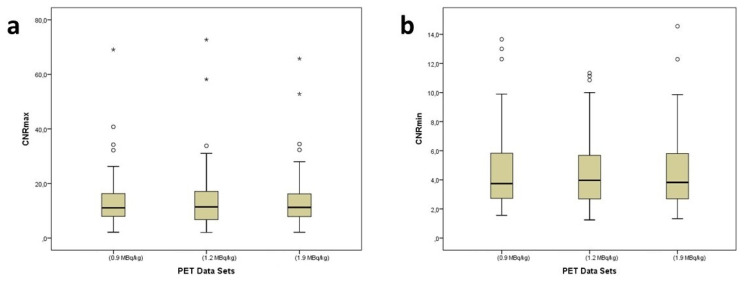
Boxplots of contrast-to-noise ratios of the lesions with maximum ^18^F-FDG uptake (CNRmax, a) and of the lesions with minimum ^18^F-FDG uptake (CNRmin, b) among PET data sets. CNRmax and CNRmin values did not show any significant differences among PET data sets (p = 0.152 and p = 0.259, respectively).

CNRmax and CNRmin values did not show any significant differences among PET data sets (p = 0.152 and p = 0.259, respectively). The median values of CNRmax and CNRmin with range were 11.2 (2.1–65.8) and 3.8 (1.3–14.6) at the original data set, 11.4 (2.1–72.7) and 3.9 (1.2–11.3) in the 1.2 MBq/kg data set, 11.1 (2.1–69.1) and 3.7 (1.6–13.7) in the 0.9 MBq/kg data set, respectively (Figures 3a and 3b). The power analysis results of the CNRmax and CNRmin for the sample size used (n = 77) were 0.93 and 0.86, respectively.

**Figure 4:**
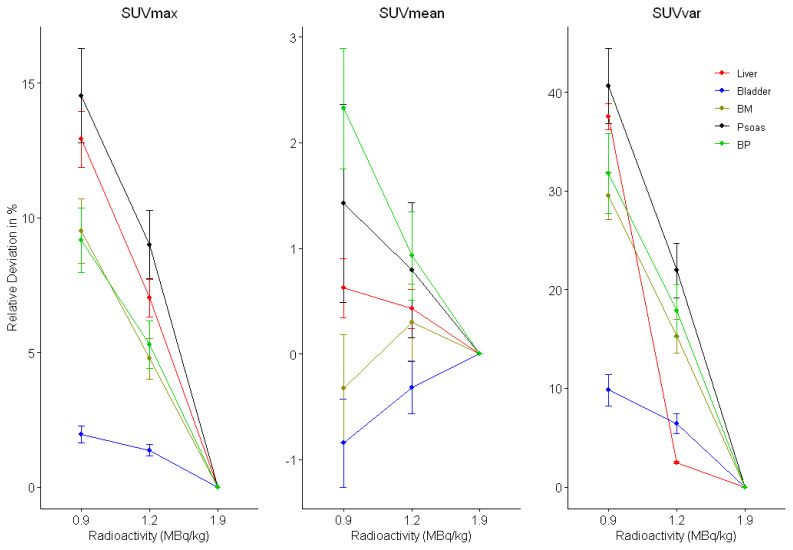
The mean relative percentage changes of SUVmax, SUVmean, and SUVvar of physiological sites in simulated low activity PET data sets compared to the original data set (BM: bone marrow; BP: mediastinal blood pool).

In physiological sites, the mean relative deviations of SUVmax, SUVmean, and SUVvar were below 10%, below 1%, and below 25%, respectively in 1.2 MBq/kg data set compared to the original data set (1.9 MBq/kg). In 0.9 MBq/kg data set compared to the original data set, the mean relative deviations of SUVmax, SUVmean, and SUVvar were above 10%, above 2%, and above 40%, respectively (Figure 4).

**Figure 5:**
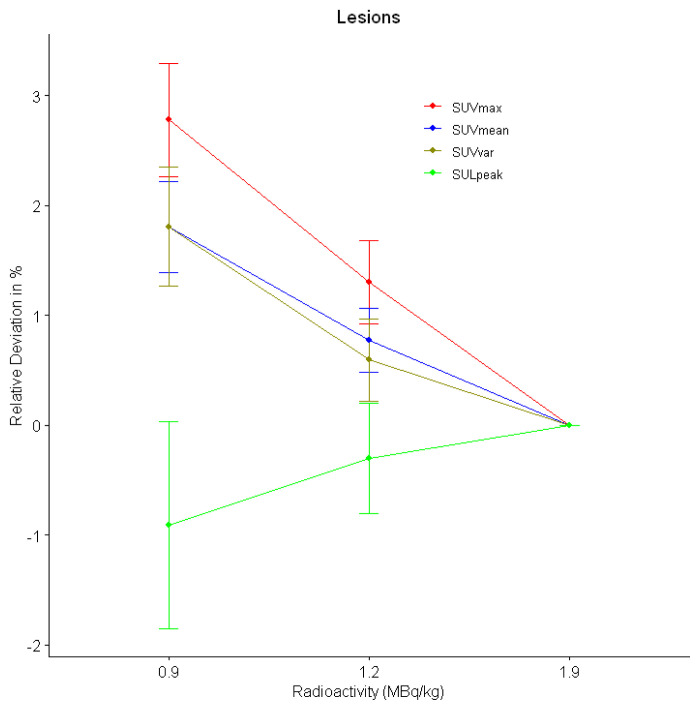
The mean relative percentage changes of SUVmax, SUVmean, SUVvar, and SULpeak of ^18^F-FDG-avid lesions in simulated low activity PET data sets compared to the original data set.

In FDG-avid lesions (n = 220), SUVmax and SUVmean were found as significantly different among PET data sets (p < 0.001 for both). In pairwise comparisons, lesion SUVmax and SUVmean in 0.9 MBq/kg data set were significantly higher compared to the original data set (p < 0.001 for both) and 1.2 MBq/kg data set (p = 0.009 and p = 0.015, respectively). However, lesion SUVmax and SUVmean did not show any significant differences between the original PET data set and 1.2 MBq/kg data set (p = 0.144 and p = 0.179, respectively). Lesion SUVvar did not show any significant differences among PET data sets (p = 0.169) (Table 1). The mean relative deviations of SUVmax was below 2% (1.3%) at 1.2 MBq/kg images and was above 2% (2.8%) at 0.9 MBq/kg images compared to original data set. The mean relative deviations of SUVmean and SUVvar were below 1% (0.8% and 0.6%, respectively) in 1.2 MBq/kg data set, and above 1% (1.8% for both) in 0.9 MBq/kg data set (Table 2) (Figure 5). The power analysis results of the SUVmax, SUVmean and SUVvar for the sample size used (n = 220) were 0.99, 0.95, and 0.83, respectively.

In FDG-avid target lesions (n = 92), SULpeak was found as significantly different between PET data sets (p = 0.003). In pairwise comparisons, SULpeak was significantly higher in the 0.9 MBq/kg data set compared to the original data set (4.66 ± 2.54 vs. 4.63 ± 2.52 respectively, p = 0.004). However, SULpeak did not show any significant difference between the original data set and the 1.2 MBq/kg data set (Table 1). The mean relative deviations of SULpeak were below 1% in 1.2 MBq/kg and 0.9 MBq/kg data sets compared to the original data set (–0.3% and –0.9%, respectively) (Table 2) (Figure 5). The power analysis result of the SULpeak for the sample size used (n = 92) was 0.97.

### 3.2. Visual evaluation of image quality

**Figure 6:**
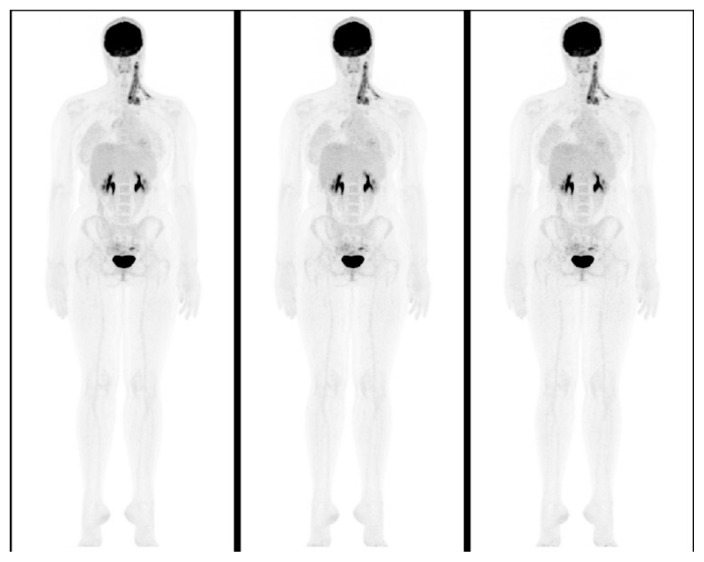
15-year-old girl with newly diagnosed Hodgkin’s lymphoma. Maximum intensity projection (MIP) images of the patient for the original data set (1.9 MBq/kg), and simulated activities of 1.2 and 0.9 MBq/kg (from left to right). SNRs were 8.0, 7.2, and 6.3, respectively. CNRmax values were 17.1, 16.3, and 16.5, and CNRmin values were 2.4, 2.4, and 2.2, respectively. In accordance with quantitative results, while there was no significant difference in the detectability of ^18^F-FDG-avid lesions, image noise, and granularity increased with decreasing tracer activities.

Figure 6 demonstrates an example for the original and simulated PET data sets. The visual image scores for the lesion detectability were 1.61 ± 0.53 for the original data set, 1.66 ± 0.51 for the 1.2 MBq/kg data set and 1.91 ± 0.65 for the 0.9 MBq/kg data set. In the visual evaluation, while there was no significant difference in the detectability of ^18^F-FDG-avid lesions between PET data sets (p = 0.233), it could be seen that image noise and granularity increased with decreasing tracer activities, more prominently in liver and MBP. The visual image scores for the general image quality and artifact, image sharpness, and image noise were found as significantly different among PET data sets (p < 0.001). The mean visual scores were 2.31 ± 0.83, 2.52 ± 0.73, and 2.95 ± 0.68 for the general image quality and artifact, 1.32 ± 0.47, 1.58 ± 0.55, and 2.28 ± 0.51 for the image sharpness, 1.1 ± 0.29, 1.64 ± 0.56, and 2.41 ± 0.55 for the image noise, respectively for original data set, 1.2 MBq/kg data set and 0.9 MBq/kg data set. In pairwise comparisons, the mean visual scores for the general image quality and artifact, image sharpness and image noise in 0.9 MBq/kg data set were significantly higher compared to the original data set and 1.2 MBq/kg data set (p < 0.001 for all). The mean visual score for the image noise was also significantly higher in 1.2 MBq/kg data set compared to the original data set (p < 0.001). However, there was no significant difference between the original data set and 1.2 MBq/kg data set in terms of general image quality/artifact and image sharpness (p = 0.181 and p = 0.077, respectively). The power analysis results of the visual scores of the general image quality/artifact, image sharpness, image noise, and lesion detectability for the sample size used (n = 77) were 1.0, 1.0, 1.0, and 0.93, respectively.

### 3.3. Texture analysis

Textural heterogeneity features showed gradually increasing values and homogeneity features showed gradually decreasing values with decreasing tracer activities. Histogram and NGTDM features did not show any significant differences among PET data sets. Entropy, Contrast, Dissimilarity heterogeneity features from GLCM were found as significantly different among data sets (p = 0.04, p = 0.002, p = 0.001, respectively). Inverse Difference Moment, which was a homogeneity feature from GLCM, was gradually decreasing with decreasing activities and showed significant difference among data sets (p = 0.001). Short Run Emphasis (SRE) and Long Run Emphasis (LRE) from GLRLM showed significant differences among PET data sets (p < 0.001, p = 0.002, respectively). While SRE showed gradually increasing values with decreasing tracer activities, LRE values showed gradually decreasing values from the original data set to the 0.9 MBq/kg data set. Large Zone Emphasis (LZE), Low Grey Level Zone Emphasis (LGLZE) and High Grey Level Zone Emphasis (HGLZE) from GLSZM were found as significantly different among PET data sets (p = 0.013, p = 0.014, p = 0.013, respectively). LZE and LGLZE showed gradually decreasing values, and HGLZE showed gradually increasing values from the original data set to the 0.9 MBq/kg data set (Table 3).

In pairwise comparisons, Entropy, Contrast, Dissimilarity, SRE, HGLZE had significantly higher, and Inverse Difference Moment, LRE, LZE, LGLZE had significantly lower values in the 0.9 MBq/kg data set compared to the original data set (p = 0.042, p = 0.002, p = 0.001, p < 0.001, p = 0.013, p < 0.001, p = 0.002, p = 0.013, p = 0.018, respectively). However, these radiomics features did not show any significant differences between the original data set and the 1.2 MBq/kg data set.

## 4. Discussion

Reducing radiation exposure in pediatric patients is of great importance because of their higher radiosensitivity and longer life expectancies than adults. One of the important potential advantages of replacing PET/CT with PET/MRI in pediatric oncology is the reduction of ionizing radiation dose. In addition to the elimination of CT-related radiation dose, the higher sensitivity of new generation PET detectors in current PET/MRI scanners and the possibility to extend PET acquisition times due to the simultaneous acquisition of PET and MRI make it possible to further reduce the radiation exposure by decreasing the injected radiotracer activities. In order to reduce the radiation exposure for children and adolescents, we have adjusted the standard injected activity as 1.9 MBq/kg and the standard PET scan duration as 5 min per bed position for the ^18^F-FDG PET/MRI protocol of our department in pediatric oncology patients since September 2017, based on the clinical experience of our department and a previous study by Gatidis et al. [8, 16]. The present study was conducted to investigate whether the injected tracer activity could be further reduced from 1.9 MBq/kg to lower levels.

In the present study, an analysis of quantitative image metrics was performed to investigate the possibility of reducing the administered tracer activity in ^18^F-FDG PET/MRI in pediatric oncology patients. SNR, CNRs, SUV metrics and textural features as well as visual scores of image quality were evaluated. For this study, the original PET data set (1.9 MBq/kg) and simulated low-activity PET images (1.2 MBq/kg and 0.9 MBq/kg data sets) were used.

Our study demonstrated that SNRs were decreasing with decreasing activities. As we expected, image noise and granularity increased gradually at lower activity images in visual evaluation, and objective SNR metrics also demonstrated the increased image noise at lower tracer activities pointing out a worsening in visual scores of image quality. However, the quantitative CNRs did not show significant difference in lower activity data sets compared to the original data set. This result may be explained by the lowest simulated tracer activity in our study. In the study by Zucchetta et al. [17], it was shown that CNR values had limited variations between simulated lower activity data sets (from 3 MBq/kg to 1.2 MBq/kg), except for the 0.6 MBq/kg data set. In our study, the lowest simulated tracer activity was 0.9 MBq/kg. This finding may also be explained by the different VOI diameters which were used for SUVvar measurements at the physiological FDG uptake sites and at the background region of CNR calculation. Although SUVvar values increased prominently in physiological sites at lower activity data sets, the VOI diameters used within these regions (1 to 3 cm) were larger than the VOI diameters used for the background standard deviation measurement in the CNR calculation (two voxels in size; 4 mm). The smaller VOIs used for the measurement of background standard deviation in the CNR calculation might have less reflected the variations of the background noise at the simulated activity amounts in our study. Despite the increased image noise in lower activity data sets, the lesion detectability was also similar among PET data sets. Nevertheless, in visual scoring of the general image quality, image sharpness and image noise, the visual image score was significantly worse in 0.9 MBq/kg data set compared to the other data sets, suggesting that the image quality was impaired at activity levels lower than 1.2 MBq/kg.

The relative changes in semiquantitative SUV parameters in physiological sites and in FDG-avid lesions were found to be lower in 1.2 MBq/kg data set compared to 0.9 MBq/kg data set. SUVmax and SUVvar parameters showed a steadily increasing deviation with decreasing tracer activities. In physiological FDG uptake sites, the highest increase was observed in the psoas muscle and liver, while the lowest increase was observed in the bladder. We observed smaller differences of SUVmean compared to SUVmax and SUVvar in physiological uptake sites. These results were similar to the results of previous studies [16, 17]. SUVmax is a single voxel value with highest maximum uptake. SUVvar is defined as the standard deviation of the SUV of all voxels within the VOI. These two parameters are expected to be more unstable and more sensitive to tracer activity reduction compared to SUVmean because of their dependence on image noise and statistical fluctuations. Since the SUVmean is the average of voxel values in a VOI, it is relatively stable against noise amplifications and fluctuations at lower count levels.

Although the relative percentage changes of SUV parameters in FDG-avid lesions were found to be very small in lower activity data sets compared to the original data set, lesion SUVmax and SUVmean values were significantly higher in 0.9 MBq/kg data set compared to the other two data sets. On the contrary, lesion SUVmax and SUVmean values did not show a statistically significant difference between the original data set and 1.2 MBq/kg data set. The SUVmax is the most frequently used semiquantitative parameter in routine clinical studies, therefore the quantitative accuracy of SUVmax may be a limiting factor in activity reduction studies. In addition to SUVmax, we obtained SULpeak metrics of target lesions according to PERCIST. SULpeak is expected to be a more stable parameter as it is the average of voxel values of a 1 cm^3^ spheric VOI centered on the highest uptake part of the tumor. As we expected, SULpeak of target lesions showed very small mean relative percentage changes (below 1%) in lower activity data sets compared to the original data set, similar to the study of Zucchetta et al. [17]. However, the SULpeak values of target lesions were significantly different in 0.9 MBq/kg data set from the other two data sets in our study. Although the relative percentage changes of lesion SUV metrics were very small among PET data sets, the statistically higher values of the lesion SUVmax, SUVmean and SULpeak in 0.9 MBq/kg data set compared to the other data sets suggest that the accuracy of PET quantification may decrease at tracer activity regimens lower than 1.2 MBq/kg.

When we review the findings in our study, it may be recommended that the reduction of injected ^18^F-FDG activity to 1.2 MBq/kg can be feasible in pediatric oncological PET/MRI. Compared to the standard injected activity of 3.7 MBq/kg in many centers, using administered ^18^F-FDG activities of 1.2 MBq/kg would be equivalent to a further reduction of radiation dose by more than 60%, when a linear relationship between administered tracer activity and absorbed dose is assumed [25]. It is also important to note that the results presented in our study were obtained on a specific PET/MRI scanner and may not be generalizable. The level of the lowest tracer activity that was proposed in our study is lower compared to the lowest tracer activity limits recommended in the previous studies. There are several reasons that could explain this difference.

Gatidis et al. performed a study with 30 whole-body FDG PET/MRI examinations of 24 pediatric oncology patients [16]. In their study, they suggested that the reduction of tracer activity to 1.5 MBq/kg was feasible without obvious diagnostic shortcomings. Zucchetta et al. evaluated 21 whole-body PET/MRI examinations of 17 patients [17]. They also showed the feasibility of the reduction of injected activity to 1.5 MBq/kg for pediatric patients. Both study used the Biograph mMR system (Siemens Healthcare, Erlangen, Germany) and this scanner is not TOF capable. Considering the similarity of PET acquisition times, the difference between the suggested minimum tracer activities in our study (1.2 MBq/kg) and these previous studies seems to be related to the use of different PET/MRI scanners. In our study, we used the GE SIGNA PET/MRI system. This scanner has higher photon sensitivity compared to Biograph mMR system [12, 26]. The SIGNA PET/MRI also has TOF capability, with a timing resolution of 385 ps, which allows for better image quality at lower count levels [19]. Therefore, further reduction in injected tracer activity was found to be possible in our study.

The study of Schmall et al. had 12 PET/MRI scans of 11 patients by using the GE SIGNA PET/MRI system [18]. Although their study showed no distinct degradation in image quality down to a linear tracer activity regimen of 1.2 MBq/kg, and the SUVmax was unstable at activity levels lower than 1.2 MBq/kg, they considered a decrease in the injected tracer activity regimen to 2.46 MBq/kg, and a second tracer regimen of 1.8 MBq/kg for high-risk patients, as a more conservative clinical approach. Considering the similarity of PET/MRI scanners, the difference between the suggested minimum tracer activities in our study and this previous study seems to be related to the use of different PET scan durations as well as different clinical approaches. While PET scan duration was 3 min per bed position in the study of Schmall et al., the standard PET scan duration was 5 min per bed position in our study. The relatively long PET acquisition times in our study can explain the reduction of tracer activity to lower levels. Based on the results of the study of Schmall et al. and our study, a tradeoff between PET scan duration and injected tracer activity can be suggested on a specific PET/MRI scanner. While shortened PET acquisiton times with relatively higher injected tracer activities may be preferred in patients with poor cooperation, the tracer activity can be reduced by extending the PET scan duration in the high-risk patient group.

Tumor heterogeneity is related to treatment resistance, disease progression, tumor invasion, relapse and metastatic spread. In recent years, it has become possible to analyze medical images to extract additional data on tumor heterogeneity. According to this approach called “Radiomics”, the information of signal heterogeneity can be translated into the knowledge of tumor heterogeneity by using the texture analysis methods [27, 28]. Textural tumor heterogeneity on FDG PET images had prognostic value in different tumor types [29, 30]. However, since the texture analysis examines the relationship and changes between voxel intensities, the quantitative values of textural features may be affected by decreased injected radiotracer activity amounts and increased image noise. In this study, we also examined the effect of activity reduction on the quantitative values of textural features of the primary tumors in FDG PET images by using simulated lower activity data sets. Histogram features did not show any significant differences among PET data sets. The histogram analysis, which is a type of first order statistics, makes a global assessment of voxel intensity within the image. Therefore, histogram textural features may be slightly affected by increased image noise at lower activity data sets. However, significant differences were found between the original data set and 0.9 MBq/kg data set in terms of higher order (GLCM, GLRLM, and GLSZM) features. In the lowest activity data set, the quantitative values of textural features indicated higher tumor heterogeneity. These radiomics features did not show any significant differences between the original data set and the 1.2 MBq/kg data set. Our results were similar to the results of the study of Branchini et al. [31]. They found that most of the histogram and textural features resulted robust till 1.2 MBq/kg in pediatric PET/MRI examinations. Our findings suggest that increasing image noise in the 0.9 MBq/kg data set significantly increases tumor heterogeneity in FDG PET images compared to other data sets. The fact that the textural features that showed significant changes with decreasing activity belong to the higher order statistics may be due to the more detailed analysis of the relationship of different voxel intensities, voxel intensity similarities, run lengths and zone sizes in these categories. These features seem to be more sensitive in reflecting the changes in image noise. In our study, it was also observed that NGTDM features were more robust parameters against to the increased image noise compared to the other higher order statistics features. The possible prognostic effect of the changes in the quantitative values of textural features due to the decreased tracer activity needs to be evaluated by using survival analysis in the future studies.

Our study had several limitations. First of all, lower activity PET images were simulated by truncating list mode PET data. Gatidis et al. used randomized undersampling method to obtain the simulated lower activity PET images in their study [16]. Compared to randomized undersampling method, simple truncation method does not preserve the temporal aspect of the PET study. Second, the results were specific to GE SIGNA PET/MRI system, and may not apply to other scanners directly. Third, administered tracer activities of 1.2 MBq/kg and 0.9 MBq/kg ^18^F-FDG were simulated in our study, and three radioactivity levels were evaluated, including the original data (1.9 MBq/kg). Including the original data, the number of activity levels which were simulated and evaluated were 7, 6, and 5 in the studies of Gatidis et al., Zucchetta et al., and Schmall et al., respectively [16–18]. In addition to the fact that the number of data sets was smaller in our study compared to the previous studies, the lowest simulated tracer activity was also at a higher level in our study than in other studies. The lowest simulated activity regimens were 0.25 MBq/kg, 0.6 MBq/kg, and 0.4 MBq/kg, respectively, in those studies. Fourth, some contemporary quantitative image quality parameters, such as mean squared error (MSE), peak signal to noise ratio (pSNR) and structural similarity index (SMI), were not analyzed in our study. Further studies with these new quantitative quality parameters are needed to evaluate the effect of activity reduction on FDG PET images. Nevertheless, our study includes the largest patient population and PET/MRI examinations with texture analysis to date, compared to the previous valuable studies in this field.

In conclusion, the results of the quantitative and the visual analyses in our study showed that the reduction of injected activity to 1.2 MBq/kg with a 5 min PET scan duration per bed position can be feasible in pediatric oncological PET/MRI, with a small relative percentage change in quantitative parameters and with similar visual image quality to the original data set. Although there were no significant differences in visual and quantitative lesion detectability between PET data sets in our study, we thought that the reduction of injected tracer activity to 0.9 MBq/kg of ^18^F-FDG could not be appropriate in order to stay in the safe range in terms of SUV metrics, radiomics quantitations and general image quality, under the conditions of the features of current PET detectors and the tolerable acquisition times. In the future, with advances in PET detector technology of PET/MRI scanners, it will be possible to further reduce injected tracer activities and minimize radiation exposure in pediatric patients.

## Figures and Tables

**Table 1:** The levels of SUVmax, SUVmean, SUVvar, and SULpeak in ^18^F-FDG-avid lesions among PET data sets.

	Original data set (1.9 MBq/kg)	1.2 MBq/kg data set	0.9 MBq/kg data set	P
SUVmax				**<0.001**
*Mean ± SD*	5.11 ± 3.44	5.15 ± 3.44	5.25 ± 3.52	
*Median with range*	3.98 (0.7–16.3)	3.93 (0.67–16.0)	4.0 (0.76–16.7)	
SUVmean				**<0.001**
*Mean ± SD*	3.11 ± 2.13	3.13 ± 2.14	3.17 ± 2.18	
*Median with range*	2.40 (0.42–10.4)	2.43 (0.43–10.3)	2.42 (0.46–10.4)	
SUVvar				0.169
*Mean ± SD*	0.72 ± 0.48	0.72 ± 0.48	0.73 ± 0.49	
*Median with range*	0.58 (0.09–2.4)	0.59 (0.11–2.7)	0.59 (0.11–2.7)	
SULpeak				**0.003**
*Mean ± SD*	4.63 ± 2.52	4.65 ± 2.56	4.66 ± 2.54	
*Median with range*	3.78 (1.12–11.3)	3.80 (1.12–11.3)	3.81 (1.17–11.4)	

The bold entries indicate a significant result.

**Table 2:** The values of relative percentage changes of SUV metrics in ^18^F-FDG-avid lesions in simulated low activity PET data sets compared to the original PET data set.

	1.2 MBq/kg data set	0.9 MBq/kg data set
SUVmax		
*Mean*	1.3	2.8
*Median with range*	0.9 (−18.1–19.6)	2.3 (−15.9–31.3)
SUVmean		
*Mean*	0.8	1.8
*Median with range*	0.5 (−13.6–13.1)	1.3 (−19.8–19.5)
SUVvar		
*Mean*	0.6	1.8
*Median with range*	0.0 (−20.9–22.2)	0.0 (−17.0–32.3)
SULpeak		
*Mean*	−0.3	−0.9
*Median with range*	0.38 (−15.1–3.5)	0.28 (−29.6–7.11)

**Table 3:** The values of FDG PET textural features of primary solid tumors among PET data sets.

	Original data set (1.9 MBq/kg)	1.2 MBq/kg data set	0.9 MBq/kg data set	P
**Histogram**				
Variance	10.2 (1.17–27.0)	10.2 (1.24–27.1)	10.14 (1.1–26.0)	0.558
Skewness	0.54 (0.32–1.2)	0.52 (0.3–1.0)	0.53 (0.34–1.1)	0.105
Kurtosis	−0.33 (–1.0–3.2)	−0.2 (−1.0–2.1)	−0.07 (−1.0–1.6)	0.076
Mean Absolute Deviation	2.7 (0.9–4.2)	2.7 (0.9–4.3)	2.7 (0.8–4.2)	0.264
Robust Mean Absolute Deviation	2.3 (0.7–3.4)	2.2 (0.7–3.2)	2.2 (0.7–3.2)	0.92
Median Absolute Deviation	2.7 (0.7–4.2)	2.7 (0.8–4.2)	2.6 (0.8–4.1)	0.472
Entropy (log 10)	1.05 (0.6–1.3)	1.06 (0.6–1.3)	1.05 (0.6–1.3)	0.779
Entropy (log 2)	3.5 (2.0–4.3)	3.5 (2.0–4.2)	3.5 (1.9–4.2)	0.779
Uniformity	0.09 (0.06–0.32)	0.09 (0.06–0.28)	0.09 (0.06–0.31)	0.92
Root Mean Square	0.01 (0.002–0.03)	0.01 (0.003–0.03)	0.01 (0.003–0.03)	0.717
**GLCM**				
Maximum	0.03 (0.01–0.22)	0.02 (0.01–0.14)	0.02 (0.01–0.16)	0.105
Average	14.3 (5.7–27.3)	14.4 (5.5–27.1)	14.2 (5.2–27.4)	0.338
Variance	10.2 (1.3–26.2)	10.2 (1.4–26.2)	10.2 (1.2–25.3)	0.558
Entropy (log 10)	2.07 (1.2–2.4)	2.08 (1.3–2.4)	2.09 (1.2–2.4)	**0.04**
Entropy (log 2)	6.85 (4.1–8.0)	6.90 (4.2–8.0)	6.93 (3.9–8.0)	**0.04**
Angular Second Moment	0.01 (0.005–0.09)	0.01 (0.005–0.08)	0.009 (0.005–0.07)	0.105
Contrast	10.1 (1.6–17.5)	10.1 (1.7–17.5)	10.4 (1.5–17.8)	**0.002**
Dissimilarity	2.47 (0.9–3.2)	2.48 (1.0–3.2)	2.53 (0.9–3.3)	**0.001**
Inverse Difference Moment	0.333 (0.3–0.6)	0.330 (0.27–0.59)	0.325 (0.27–0.6)	**0.001**
**GLRLM**				
SRE	0.910 (0.82–0.93)	0.912 (0.82–0.94)	0.915 (0.81–0.94)	**<0.001**
LRE	1.46 (1.3–2.2)	1.45 (1.3–2.1)	1.43 (1.3–2.3)	**0.002**
GLNU	88.5 (31.6–2166)	90.4 (30.2–2204)	101.7 (32.3–2233)	0.205
RLNU	667.2 (140.9–10916)	667.9 (154.6–10413)	703.2 (185.9–11425)	0.472
**NGTDM**				
Coarseness	0.008 (0.001–4.7)	0.009 (0.001–4.44)	0.009 (0.001–4.1)	0.338
Contrast	0.108 (0.03–0.26)	0.107 (0.04–0.24)	0.111 (0.05–0.24)	0.558
Busyness	0.98 (0.41–12.9)	0.95 (0.57–12.4)	0.89 (0.56–12.6)	1.0
Complexity	134.6 (15.4–746.3)	134.2 (11.0–780.4)	133.1 (10.3–710.4)	0.338
**GLSZM**				
SZE	0.504 (0.39–0.6)	0.534 (0.23–0.6)	0.545 (0.40–0.61)	0.558
LZE	315.9 (48.1–61958)	269.9 (36.8–62047)	263.6 (29.3–42494)	**0.013**
LGLZE	0.0076 (0.002–0.05)	0.0070 (0.002–0.04)	0.0065 (0.002–0.03)	**0.014**
HGLZE	165.1 (30.4–710)	183.8 (38.3–693.8)	190.1 (42.5–724.9)	**0.013**
ZSNU	49.2 (3.3–326.1)	51.3 (1.4–336.9)	55.2 (2.1–393.9)	0.097

The bold entries indicate a significant result.

The results were displayed as median with ranges.

Abbreviations: GLCM: grey level cooccurence matrix, GLRLM: grey level run length matrix, SRE: short run emphasis, LRE: long runs emphasis, GLNU: grey level nonuniformity, RLNU: run length nonuniformity, NGTDM: neighborhood grey-tone difference matrix, GLSZM: grey-level size zone matrix, SZE: small zone emphasis, LZE: large zone emphasis, LGLZE: low grey level zone emphasis, HGLZE: high grey level zone emphasis, ZSNU: zone size nonuniformity.
